# Impaired Neurovascular Function Underlies Poor Neurocognitive Outcomes and Is Associated with Nitric Oxide Bioavailability in Congenital Heart Disease

**DOI:** 10.3390/metabo12090882

**Published:** 2022-09-19

**Authors:** Vanessa J. Schmithorst, Phillip S. Adams, Daryaneh Badaly, Vincent K. Lee, Julia Wallace, Nancy Beluk, Jodie K. Votava-Smith, Jacqueline G. Weinberg, Sue R. Beers, Jon Detterich, John C. Wood, Cecilia W. Lo, Ashok Panigrahy

**Affiliations:** 1 Department of Pediatric Radiology, UPMC Children’s Hospital, Pittsburgh, PA 15224, USA; 2 Department of Pediatric Anesthesiology, UPMC Children’s Hospital, Pittsburgh, PA 15224, USA; 3 Learning and Development Center, Child Mind Institute, New York, NY 10022, USA; 4 Department of Bioengineering, University of Pittsburgh, Pittsburgh, PA 15213, USA; 5 Heart Institute, Children’s Hospital Los Angeles, Los Angeles, CA 90027, USA; 6 Department of Pediatric Cardiology, UPMC Children’s Hospital, Pittsburgh, PA 15224, USA; 7 Department of Psychiatry, University of Pittsburgh School of Medicine, Pittsburgh, PA 15213, USA; 8 Department of Developmental Biology, University of Pittsburgh, Pittsburgh, PA 15213, USA; 9 Department of Radiology, University of Pittsburgh School of Medicine, Pittsburgh, PA 15213, USA

**Keywords:** congenital heart disease, neuro-vascular function, neurocognition, nitric oxide bioavailability

## Abstract

We use a non-invasive MRI proxy of neurovascular function (pnvf) to assess the ability of the vasculature to supply baseline metabolic demand, to compare pediatric and young adult congenital heart disease (CHD) patients to normal referents and relate the proxy to neurocognitive outcomes and nitric oxide bioavailability. In a prospective single-center study, resting-state blood-oxygen-level-dependent (BOLD) and arterial spin labeling (ASL) MRI scans were successfully obtained from 24 CHD patients (age = 15.4 ± 4.06 years) and 63 normal referents (age = 14.1 ± 3.49) years. Pnvf was computed on a voxelwise basis as the negative of the ratio of functional connectivity strength (FCS) estimated from the resting-state BOLD acquisition to regional cerebral blood flow (rCBF) as estimated from the ASL acquisition. Pnvf was used to predict end-tidal CO2 (P_ET_CO2) levels and compared to those estimated from the BOLD data. Nitric oxide availability was obtained via nasal measurements (nNO). Pnvf was compared on a voxelwise basis between CHD patients and normal referents and correlated with nitric oxide availability and neurocognitive outcomes as assessed via the NIH Toolbox. Pnvf was shown as highly predictive of P_ET_CO2 using theoretical modeling. Pnvf was found to be significantly reduced in CHD patients in default mode network (DMN, comprising the ventromedial prefrontal cortex and posterior cingulate/precuneus), salience network (SN, comprising the insula and dorsal anterior cingulate), and central executive network (CEN, comprising posterior parietal and dorsolateral prefrontal cortex) regions with similar findings noted in single cardiac ventricle patients. Positive correlations of Pnvf in these brain regions, as well as the hippocampus, were found with neurocognitive outcomes. Similarly, positive correlations between Pnvf and nitric oxide availability were found in frontal DMN and CEN regions, with particularly strong correlations in subcortical regions (putamen). Reduced Pnvf in CHD patients was found to be mediated by nNO. Mediation analyses further supported that reduced Pnvf in these regions underlies worse neurocognitive outcome in CHD patients and is associated with nitric oxide bioavailability. Impaired neuro-vascular function, which may be non-invasively estimated via combined arterial-spin label and BOLD MR imaging, is a nitric oxide bioavailability dependent factor implicated in adverse neurocognitive outcomes in pediatric and young adult CHD.

## 1. Introduction

Congenital heart disease (CHD) patients demonstrate early neurodevelopmental deficits that are associated with neurovascular abnormalities [1], including impaired autoregulation [2], suggesting underlying neurovascular abnormalities in early fetal and infant brain development. As the cardiovascular disease burden shifts in older CHD patients from factors associated with the heart disease itself to acquired cardiovascular co-morbidities, there is an increased scale of neurovascular disease [3]. As such, pediatric and adult CHD patients are at high risk of stroke [4,5,6] and microvascular ischemic disease. We have recently documented that patients with unfavorable CHD physiological features known to be associated with increased acquired brain injury demonstrate low nasal nitric oxide (nNO) [7]. We further demonstrated that low nNO, a proxy of nitric oxide bioavailability, was also predictive of poor cardiac related outcomes in CHD patients, including that of heart failure and low oxygen saturations [7]. While cerebral NO has been implicated in cerebrovascular autoregulation [8,9,10], it is unknown if reduced nitric oxide bioavailability is associated with increased risk of neurovascular disease in CHD patients.

These considerations lead us to hypothesize that the neurocognitive deficits frequently seen in pediatric and young adult CHD patients [11,12,13,14,15] may be partly accounted for by impairments in neurovascular function (nvf) and reduced nitric oxide bioavailability. We here define nvf as the capability of the vasculature to provide for metabolic demand in the resting-state. NVF is distinguished from cerebrovascular reserve/reactivity (CVR), which refers to the capacity in the vasculature for increased cerebral blood flow (CBF) over the resting-state [16].

We have developed a non-invasive MRI proxy for nvf (Pnvf) as the ratio of baseline CBF to baseline cerebral metabolic rate of oxygen (CMRO2). The higher this ratio, the more the vasculature over supplies for the baseline metabolic demand, which we hypothesize renders the vasculature more resilient to abnormalities and disease, and thus results in improved neurocognitive outcomes. The technique uses resting-state blood-oxygen-level-dependent (BOLD) and arterial spin labeling (ASL) brain MRI acquisitions that are readily obtainable in any clinical or research population able to hold still for the duration of the scans, and that often already comprise part of either the clinical or research protocol. Regional CBF may be estimated from the ASL acquisitions [17]. From the BOLD acquisitions, we use functional connectivity strength (FCS) as a proxy for baseline CMRO2, as resting-state BOLD cannot provide an estimate of absolute neuronal or metabolic activity but only of synchronization of neuronal activity. Functional connectivity and CMRO2 have been found to be highly correlated, with certain regions of the brain forming a densely interconnected “rich-club” with high metabolic demand [18,19,20]. Additional support for use of FCS as a proxy comes from the fact that not only metabolism but also regional FCS increases relative to baseline in response to increased cognitive demand [21], indicating a “connectivity demand” for cognitive activity associated with metabolic demand. Although it is also possible to directly estimate baseline CMRO2 using MRI [22,23,24], it requires significantly longer scan times, making the technique unsuitable for many clinical and research populations.

In this prospective study, therefore, we compare Pnvf in a population of children and young adults with CHD to normal referents, and associate Pnvf with neurocognitive outcome as assessed via the NIH Toolbox in order to investigate the hypotheses that differences in Pnvf partially explain differences in neurocognitive outcomes. We also evaluated for associations with nasal nitric oxide (nNO) as previously measures in CHD patients [7], given that cerebral NO is implicated in cerebrovascular autoregulation [8,9,10] and nNO is a marker for NO bioavailability. By examining associations with nNO, we could consider the hypothesis that neurocognitive outcomes may also be related to reduced NO bioavailability that would, in turn, be reflected in reduced nNO and impaired autoregulation.

## 2. Methods

### 2.1. Participants

Participants were prospectively recruited from childhood and young adulthood (6–25 years of age) with the use of flyers/advertisement posted in clinics, on social media, and through the University of Pittsburgh Clinical Translational Research Registry. Exclusion criteria included (a) severe intellectual disability, (b) clinically diagnosed genetic syndrome associated with neurocognitive impairment as identified from the medical record, and (c) standard MRI exclusion criteria such as metallic implants. Informed consent was obtained according to the standard procedures in use at the University of Pittsburgh from all participants aged 18 and over. Consent from one parent and assent was obtained for all participants under the age of 18. All participants were scanned using a 3 Tesla Siemens Skyra system (Munich, Germany) at UPMC Children’s Hospital of Pittsburgh using a 32-channel head coil. 

### 2.2. Pnvf Estimation: Overview

**Figure 1:**
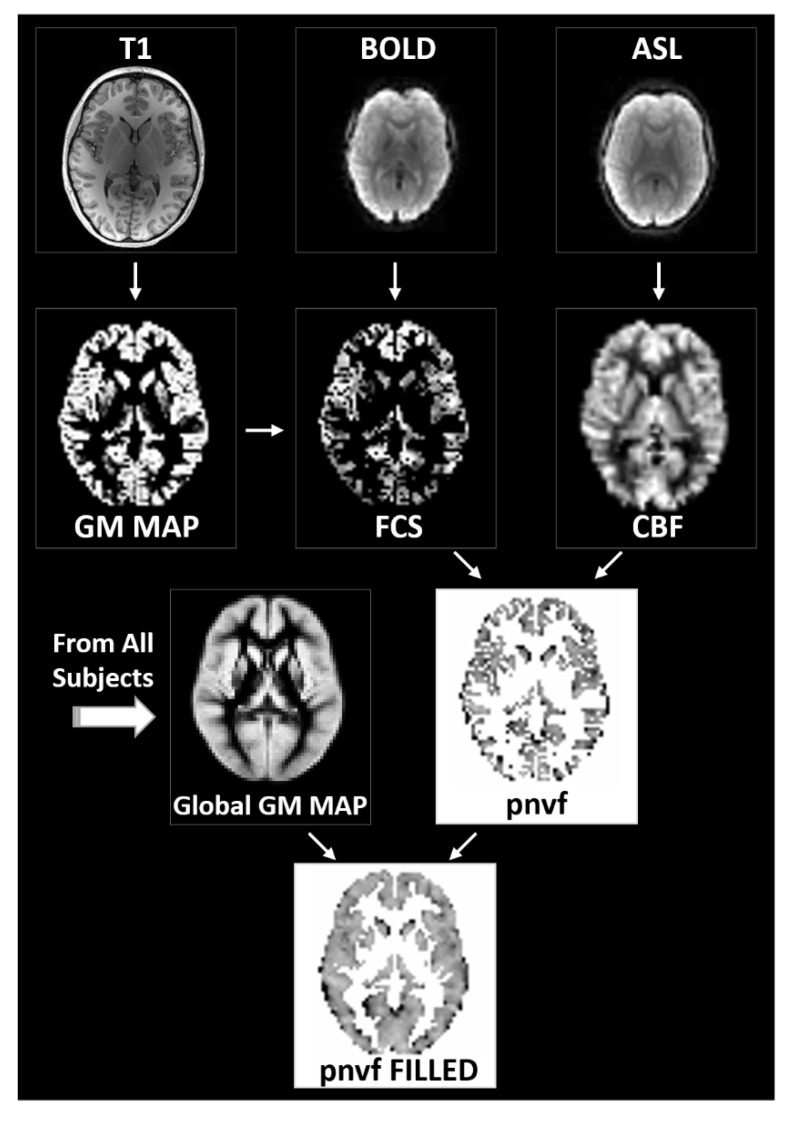
Flowchart of the data processing routines for the estimation of the proxy for neurovascular function (Pnvf). Gray matter voxels are determined via segmentation of the whole-brain anatomical T1 images (**left**). From the BOLD resting-state data, functional connectivity strength (FCS) is computed voxelwise as the average correlation coefficient between a gray matter voxel time course and the time courses of all other gray matter voxels (**center**). From the ASL data, cerebral blood flow (CBF) maps are computed based on the difference between the labeled and unlabeled acquisitions (**right**). All maps are transformed into standardized Montreal Neurological Institute (MNI) space. Pnvf is computed on a voxelwise basis as the negative of the FCS/CBF ratio for all voxels in the global gray matter mask; missing values are filled in via trilinear interpolation (**bottom**).

An overview of the process to estimate Pnvf is given in Figure 1 and described in detail below. The whole-brain images were segmented into gray matter, white matter, and cerebrospinal fluid (CSF), and the gray matter images were transformed into standardized Montreal Neurological Institute (MNI) space. After transformation of the resting-state BOLD acquisitions into MNI space, the FCS in a gray matter voxel was computed as the averaged threshold correlation coefficient between its time course and the time courses of all other gray matter voxels. From the pCASL acquisitions, CBF is computed from the contrast created via “labeling” or inverting incoming blood from the carotid arteries prior to perfusing into the tissue; the CBF maps were likewise transformed into MNI space. Although Pnvf could then be estimated on a voxelwise basis as the ratio of CBF/FCS, due to noise considerations (FCS being a noisier measurement compared to CBF), we preferred to use the negative ratio of FCS/CBF. Between-participant analyses were performed for all voxels in a global gray matter mask; missing data in a participant (e.g., due to imperfect spatial registration) were filled in via trilinear interpolation from neighboring voxels.

### 2.3. MRI Acquisitions

Whole-brain anatomical images were obtained using a T1-weighted MP-RAGE sequence with 1 mm isotropic resolution. Images were obtained on Seimens Skyra, manufactured in Erlangen, Munich, Germany. Pseudo-continuous ASL (pCASL) imaging was performed using a 2D GE-EPI readout with ascending slice order acquisition. Imaging parameters were matrix size = 64 × 64, GRAPPA = 2, in-plane resolution = 4 mm, slice thickness = 5 mm, 32 acquired slices, TR = 4100 ms, TE = 12 ms, 45 label/control pairs acquired. Labeling parameters were labeling duration = 1500 ms, post-inversion delay (initial) = 1200 ms, RF (Hanning shape) pulse duration = 330 μs, duration between RF pulses = 1 ms, mean B1 = 1.5 μT, mean gradient during labeling = 6 mT/m, mean gradient during control = 0 mT/m. Resting-state BOLD imaging was performed using 2D GE-EPI. Imaging parameters were matrix size = 64 × 64, GRAPPA = 2, in-plane resolution = 4 mm × 4 mm, slice thickness = 4 mm, 36 acquired slices, TR = 2000 ms, TE = 32 ms. 150 frames were acquired for each run (5 min total acquisition time).

### 2.4. Anatomical Pre-Processing

The anatomical T1-weighted images were segmented into gray matter, white matter, and CSF probability maps using routines in SPM8 (Wellcome Dept. of Cognitive Neurology, London, UK) and the template prior images provided in SPM8. Using the affine transformation output by the segmentation, the gray matter probability maps were spatially normalized into standardized (MNI) space with 3 mm isotropic resolution. Voxels in the cerebellum were identified using the Automated Anatomical Labeling (AAL) template [25] and removed from further analysis.

### 2.5. ASL Pre-Processing and CBF Estimation

CBF maps were computed for each participant in the following manner. The raw ASL images were motion-corrected using an affine transformation. A voxelwise GLM was used with control and control-label as parameters of interest, and motion and drift parameters as nuisance covariates. Fractional change maps were computed as (control-label)/control. CBF maps were then computed using the two-compartment model [26,27] with literature values [28] used for arterial T1 and T1 of gray matter and arterial and tissue transit times, taking into account the fact that the true post-inversion delay varies as a function of slice location.

The reference EPI images were segmented into gray matter, white matter, and CSF using routines in SPM8 and the gray matter images were transformed into MNI space using the provided gray matter template. A study-specific template was then created via averaging the transformed gray matter images and the transformation repeated. The CBF maps were then transformed into MNI space using the same transformation and resampled to 3 mm isotropic resolution.

### 2.6. BOLD Pre-Processing and FCS Computation

The pre-processing methodology very closely follows that described in previously published work [29,30] and minimized the risk of spurious correlations due to participant motion. The raw BOLD images were slice-timing corrected, and then motion corrected using an affine transformation. The reference EPI images were normalized into MNI space using an affine transformation and the provided EPI template in SPM8. A study-specific template was created via averaging the transformed EPI images and the transformation repeated. The motion-correction transformation and the spatial normalization transformation were combined into a single step to avoid repeated interpolations, and each run was normalized to a global average of 1000, resampled to 3 mm isotropic resolution.

The parameters of framewise displacement (FD) and spatial standard deviation of successive difference images (DVARS) were computed, and frames were censored at a threshold of FD > 0.2 mm or DVARS > 25. Individual runs were discarded if there were less than 50 frames remaining after censoring, and participants were also removed from analysis if there were less than 150 total frames remaining across both runs. Nuisance regressors (including motion correction parameters and drift) were regressed out from each voxel time course, and then each voxel time course was band-pass filtered with a passband of 0.009 Hz < *f* < 0.08 Hz.

FCS maps were computed for each participant as follows: FCS for a gray matter voxel was defined as the average of the Pearson correlations between its voxel time course and all other voxel time courses in the gray matter, with negative correlations being set to zero. Cerebral gray matter voxels were determined as those with a >66% value from the probability map obtained from the segmentation of the anatomical T1-weighted images. FCS was computed individually for each BOLD acquisition.

### 2.7. Pnvf Computation

Pnvf was computed on a voxelwise basis as the negative of the ratio of FCS to CBF. To improve robustness, a minimum CBF value was used of 20 mL/min/100 g (i.e., well below typical perfusion values in gray matter); voxels with less than the minimum CBF were set to that value. The final value of Pnvf was the mean of the two values computed from each of the BOLD runs. As sulci did not align perfectly across participants due to spatial registration errors, a global gray mask was constructed via participant-wide averaging of the spatially normalized gray matter probability maps; voxels were retained in the mask with average 50% probability or higher. Missing data (e.g., voxels in the global mask but not in an individual’s gray matter) were filled in via trilinearly interpolating from neighboring data points.

### 2.8. Nasal Nitric Oxide Analysis

We acquired nNO values from a subset of the participants (*n* = 44) using tidal breath sampling with a CLD 88sp NO analyzer obtained via low continuous suction at a rate of 0.3 L/min from each naris using a nasal olive sampling cannula during tidal breathing. Each naris was sampled once for 50 s. Five peak inflections from nNO concentration curve were analyzed against the sampling flow and averaged to yield a final value in nL/min. The GLM analysis of nNO vs. Pnvf was performed on a voxelwise basis; participant age at time of nNO data collection (which differed slightly from age at scan for some participants) was included as an additional covariate. Since the nNO analysis contained fewer participants, a more relaxed threshold of 200 contiguous voxels with Z > 2.0 was used; this relaxed threshold nevertheless also corresponded to FDR-corrected q < 0.05. Similarly, a mediation analysis was performed with CHD status as independent variable, nNO as mediator, and Pnvf as dependent variable in order to investigate whether different Pnvf levels in CHD patients may be partly explained by differences in NO bioavailability.

### 2.9. NIH Toolbox Cognitive Battery

Participants were administered the NIH Toolbox Cognitive Battery, a brief, computerized test battery with well-established construct validity, test–retest reliability, and developmental sensitivity in adult and pediatric samples [31,32]. The Cognitive Battery consists of the following seven subtests that measure the following cognitive abilities (in parentheses): Oral Reading Recognition (language/oral reading skill), Picture Sequence Memory (episodic memory), Pattern Comparison (processing speed), Dimensional Change Card Sort (executive Function/cognitive flexibility), List Sorting Working Memory (working memory), Picture Vocabulary (language/vocabulary knowledge), and Flanker (executive function/inhibitory control and visual attention). Based on manualized procedures, three age-normalized composite scores (mean = 100, σ = 15) were computed: Total Cognition (including all subtests), Crystallized Cognition (Oral Reading Recognition and Picture Vocabulary), and Fluid Cognition (Picture Sequence Memory, Pattern Comparison, Flanker, Dimensional Change Card Sort, List Sorting Working Memory).

### 2.10. Theoretical Modeling

We estimated time courses for P_ET_CO2 derived from the resting-state BOLD acquisitions alone using a previously published method [33] that consists of frequency filtering the global BOLD signal in gray matter with a passband of 0.02 Hz < *f* < 0.04 Hz, the frequency band found to correlate the most with measured P_ET_CO2. We also used our Pnvf maps to construct an alternative estimator of P_ET_CO2, based on the idea that the BOLD signal in voxels with greater nvf should be more greatly influenced by changes in P_ET_CO2. We zero-meaned the Pnvf values in gray matter, computed a weighted (e.g., with zero-meaned Pnvf values) average of the resting-state BOLD time courses, and frequency filtered as above. To make the test even more stringent, we used the first resting-state BOLD run with Pnvf computed from the second run, and vice versa, and averaged the results. We then correlated the two estimators of P_ET_CO2, testing the null hypothesis of zero correlation, which would be expected if there were no relation between Pnvf and BOLD signal changes due to changes in P_ET_CO2.

### 2.11. Correlational Analyses

In order to investigate associations between Pnvf, CHD status, and neurocognitive outcomes, the following General Linear Model analyses were performed on a voxelwise basis: (1) CHD status vs. Pnvf; (2) CHD status-X-age interaction on Pnvf; (3) NIH Toolbox composite scores (Total Cognition, Crystallized Cognition, and Fluid Cognition) vs. Pnvf. Participant gender and age at scan were included as covariates in all analyses; CHD status was also included as a covariate for analysis 2. T-score maps were converted into Z-score maps and 3D Gaussian filtered with σ = 4 mm; the autocorrelation of the residuals were used to construct appropriate “noise” maps [34] with appropriate amounts of endogenous and exogenous smoothness. From those noise maps, a threshold of 125 contiguous voxels with Z > 2.5 was shown to correspond to a False Discovery Rate (FDR)-corrected q < 0.05.

### 2.12. Mediation Analysis

To more closely investigate whether reduced Pnvf could indirectly effect adverse neurocognitive outcomes in CHD patients, a mediation analysis [35] was performed with CHD as an independent variable, Pnvf as a mediator, and NIH Toolbox scores as dependent variables. Participant gender and age at scan were included as covariates in all analyses. Bootstrapping (1000 repetitions) was used to generate statistical inference (*p*-value) at the voxel level for the mediation analysis [36] using bias-corrected and accelerated conference intervals [37,38]; p-scores were then converted into Z-scores to construct a voxelwise map, 3D Gaussian filtered with σ = 4 mm. The same thresholds for statistical significance were used here as for the correlational analyses.

### 2.13. Demographics Analysis

Additionally, we looked at correlations of Pnvf with age and gender (including CHD status as a covariate). We would expect a global decrease in Pnvf with age (as it is known the brain loses resilience to insult with age). Through the developmental period up until the onset of aging, girls and women are in general more resilient to neurologic insult, with lower incidence of neuropathologies including autism spectrum disorder [39], ADHD [40,41], schizophrenia [42], intellectual disability [43], and dyslexia [44]. As such, girls and women may display higher Pnvf globally or in regionally specific areas.

### 2.14. Test–Retest Reliability

Since two estimates of Pnvf were available (e.g., from each of the two BOLD runs), we investigated test–retest reliability by computing, on a voxelwise basis, the Pearson correlation coefficient between values from run 1 and run 2. 

## 3. Results

### 3.1. Recruitment and Demographics

The study included 139 participants who were recruited for the study and for whom some imaging data were available. For 103 of these participants, two resting-state BOLD runs and one ASL acquisition were acquired, as the ASL acquisition was added to the research protocol later during the study. Resting-state data from three participants were discarded due to technical problems (i.e., excess susceptibility artifacts). Of the remaining participants, 88 had two acceptable resting-state BOLD datasets using the stringent criteria for motion censoring detailed below. Of these participants, 85 were administered the NIH Toolbox Cognitive Battery.

Demographic information and neurocognitive test results for all participants for whom imaging data were successfully acquired are summarized in Table 1. There was no significant difference in gender or maternal education between CHD participants and normal referents; the CHD participants were slightly older than the normal referents and hence age was controlled for in all analyses. Regarding neurocognitive data, the NIH Toolbox Total Composite Cognition score was lower in CHD participants compared to normal referents at a trend level; however, the CHD group was in a neurocognitively normal range on average. The CHD population was heterogeneous with respect to heart lesion type (Table 2). Repeat scans were obtained from three participants. All data were included in the analysis; the degrees of freedom was appropriately reduced by three in all statistical analyses to avoid underestimation of parameter standard errors due to non-independence of error variance.

### 3.2. Theoretical Modeling

Using Fisher’s Z transformation, the Z-transformed correlation coefficient between the two estimators of P_ET_CO2 (mean +/− SEM) is 12.04 +/− 0.74 (*p* < 0.001), indicating to a very high degree of significance that Pnvf is associated with P_ET_CO2-induced BOLD signal changes and confirming the construct validity of our metric.

### 3.3. Associations of Pnvf with CHD and Neurocognitive Outcome

**Figure 2:**
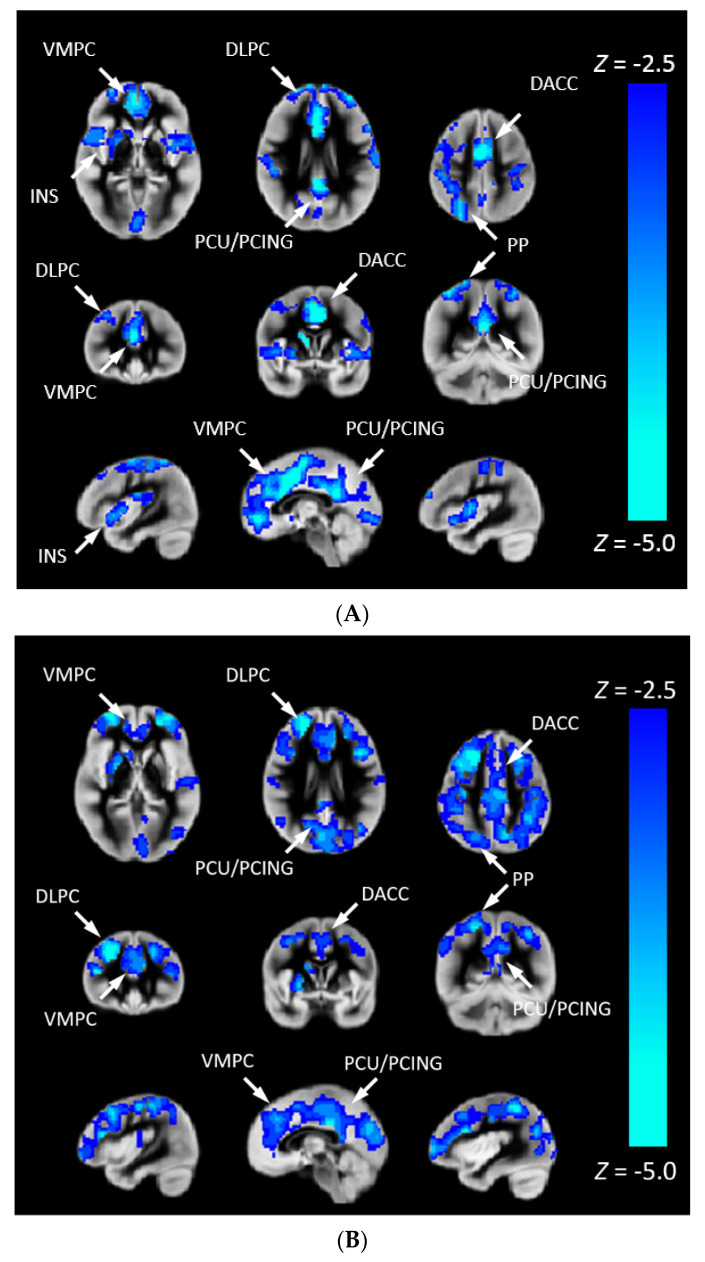
(**A**) Pnvf is reduced in children and younger adults with congenital heart disease (CHD) compared to normal referents. (**B**) Pnvf is reduced in children and younger adults with single ventricle congenital heart disease (CHD) compared to bi-ventricular congenital heart disease. Regions with decreased Pnvf (controlling for age and gender) are mainly concentrated in default mode network (DMN), central executive network (CEN), and salience network (SN) regions. All regions significant at FDR-corrected q < 0.05. Abbreviations: Ventrolateral medial prefrontal cortex (VMPC), Insula (INS), Dorsolateral prefrontal cortex (DLPC), Posterior Cingulate (PCING), Precuneus (PCU), Dorsal Anterior Cingulate Cortex (DACC), Posterior Parietal (PP).

**Figure 3:**
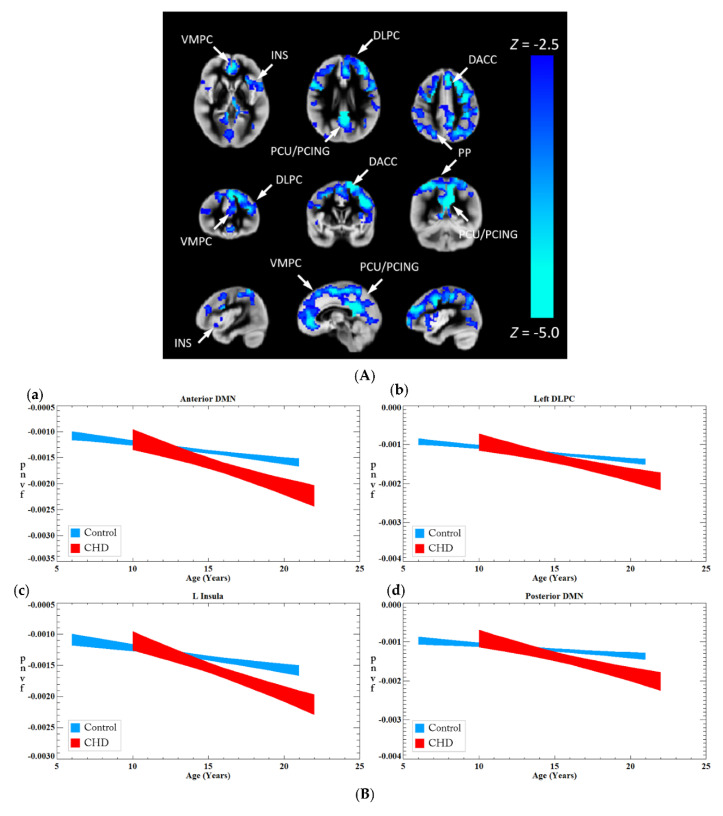
(**A**) A CHD-X-age interaction on Pnvf is present in children and younger adults. For illustrative purposes, the trajectories of CHD patients and normal referents were computed separately for the ROIs shown to exhibit a significant interaction from the voxel-wise analysis depicted in (**B**). These plots show the fitted trajectories +/− standard error of the fitted value (confidence interval = 67%). The plots show that, while at the earliest age (5 years) CHD patients have either the same or slightly higher Pnvf, the trajectory is such that they have substantially lower Pnvf by young adulthood. Regions with a negative CHD-X-age interaction on Pnvf (controlling for gender) are mainly concentrated in default mode network (DMN), central executive network (CEN), and salience network (SN) regions. The beta–age interaction values are as follows: Anterior DMN −0.20 (0.09) L Insula −0.19 (0.09), Left DLPC −0.19 (0.09), Posterior DMN −0.24 (0.09), R Insula −0.20 (0.09) All regions significant at FDR-corrected q < 0.05 as see as blue voxel in (**B**). (**B**) Comparison of Pnvf as a function of age between CHD (red) and controls (blue) in specific regions shown in Figure 3: (**a**) dorsolateral prefrontal region; (**b**) insular cortex; (**c**) anterior cingulate; and (**d**) posterior cingulate/precuneus; indicating that the Pnvf differences are more prevalent at older ages. (See Figure Legend for (**A**) for more detail). Abbreviations: default mode network (DMN), Ventrolateral medial prefrontal cortex (VMPC), Insula (INS), Dorsolateral prefrontal cortex (DLPC), Posterior Cingulate (PCING), Precuneus (PCU), Dorsal Anterior Cingulate Cortex (DACC), Posterior Parietal (PP).

We investigated the association between CHD and Pnvf (Figure 2). CHD patients showed lower Pnvf mainly in default mode network (DMN) regions (ventromedial prefrontal, posterior cingulate/precuneus, angular gyrus), salience network (SN) regions (insula, dorsal anterior cingulate) and central executive network (CEN) regions (dorsolateral prefrontal, posterior parietal) (Figure 2A). A similar relationship was noted in single ventricle patient compared to bi-ventricular patients (Figure 2B). A negative CHD-X-age interaction was also seen in these regions (Figure 3A) and subsequent ROI analyses confirmed Pnvf differences with CHD were present to a greater extent at older ages (Figure 3B).

**Figure 4:**
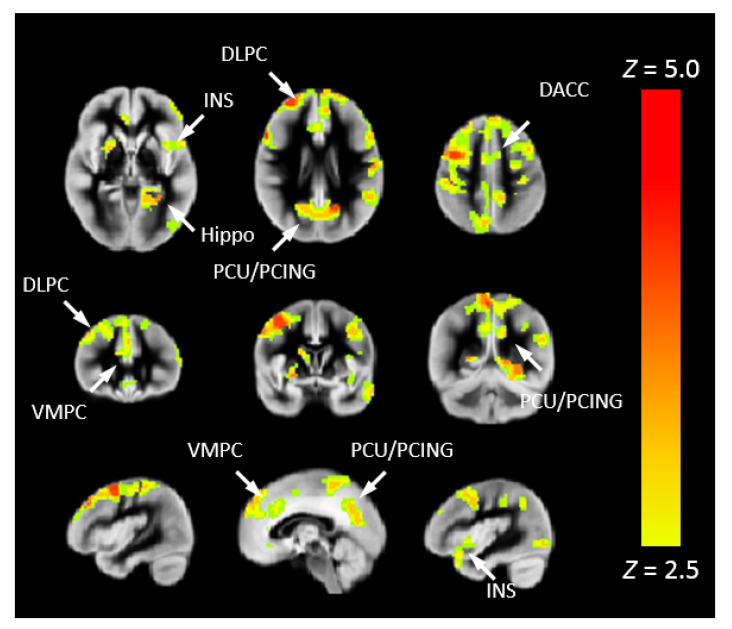
Pnvf positively correlates with neurocognitive outcomes in children and younger adults with CHD and normal referents. Regions with a positive correlation of Pnvf with the NIH Toolbox Total Cognition composite score (controlling for age, gender, and CHD status) are mainly concentrated in DMN, SN, and CEN regions as well as hippocampus and putamen. All regions depicted with yellow voxels demonstrate significant positive correlation with neurocognitive outcomes at FDR-corrected q < 0.05. Abbreviations: Ventrolateral medial prefrontal cortex (VMPC), Insula (INS), Dorsolateral prefrontal cortex (DLPC), Posterior Cingulate (PCING), Precuneus (PCU), Dorsal Anterior Cingulate Cortex (DACC), Posterior Parietal (PP).

**Figure 5:**
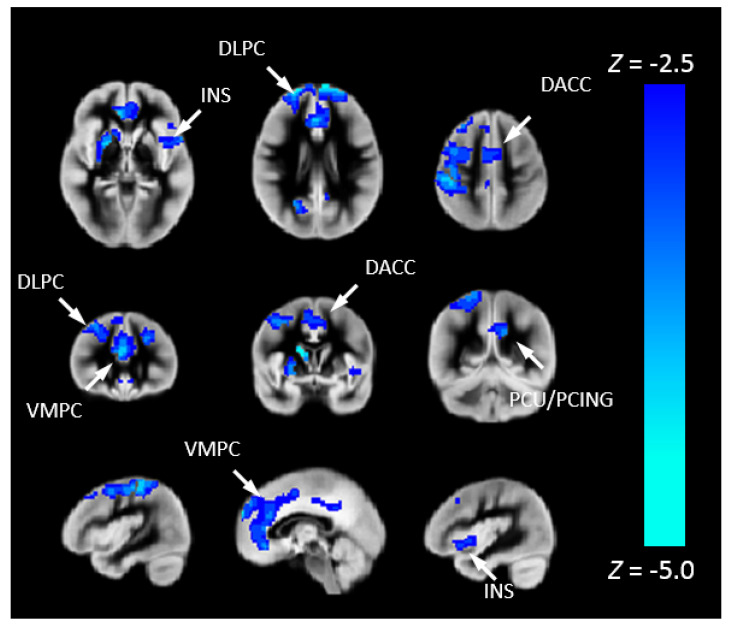
Reduced Pnvf mediates adverse neurocognitive outcome in children and younger adults with CHD. Regions with a negative indirect effect with CHD status the independent variable, NIH Toolbox Total Cognition Composite score the dependent variable, and Pnvf the mediator (controlling for age and gender) are mainly concentrated in DMN, SN, and CEN regions. All significant regions demonstrating statistically significant mediations (indirect effects) are depicted with blue voxels at FDR-corrected q < 0.05. Abbreviations: Ventrolateral medial prefrontal cortex (VMPC), Insula (INS), Dorsolateral prefrontal cortex (DLPC), Posterior Cingulate (PCING), Precuneus (PCU), Dorsal Anterior Cingulate Cortex (DACC), Posterior Parietal (PP).

To investigate the risk of cognitive changes in CHD, we correlated Pnvf with NIH Toolbox scores. A positive correlation with the Total Cognition composite score was also seen in DMN, CEN, and SN regions as well as the right hippocampus (Figure 4). Mediation analyses allowed us to investigate these results in further detail. Thus, we investigated whether neurocognitive outcomes in CHD patients are mediated by Pnvf. For Total Cognition, we found significant negative indirect effects in the DMN, CEN, and SN (Figure 5). Even stronger effects were found for Crystallized Cognition (Supplementary Material).

### 3.4. nNO Analysis

**Figure 6:**
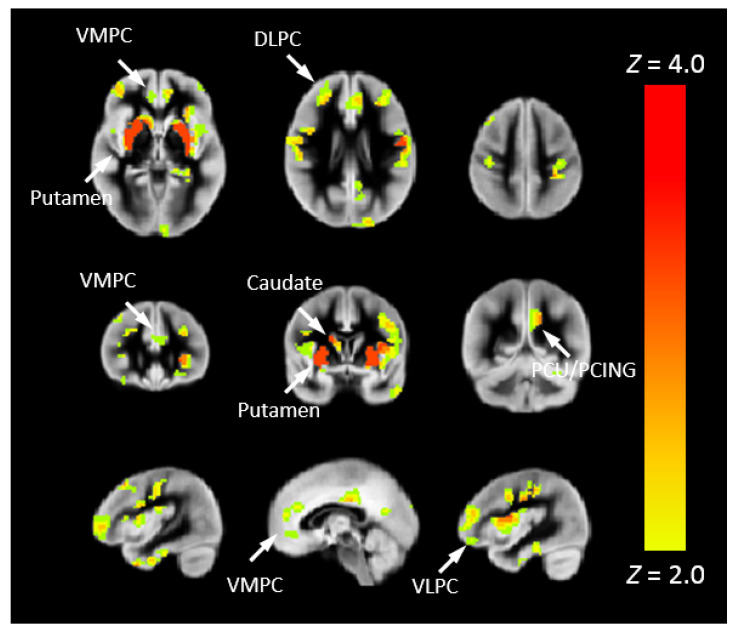
Nasal nitric oxide (nNO) positively correlates with Pnvf in children and younger adults with CHD and normal referents. Regions with a positive correlation of Pnvf with nNO (controlling for age and gender) are mainly concentrated in DMN and CEN regions as well as the putamen. All regions significant at FDR-corrected q < 0.05. Abbreviations: Ventrolateral medial prefrontal cortex (VMPC), Insula (INS), Dorsolateral prefrontal cortex (DLPC), Posterior Cingulate (PCING), Precuneus (PCU), Dorsal Anterior Cingulate Cortex (DACC), Posterior Parietal (PP).

**Figure 7:**
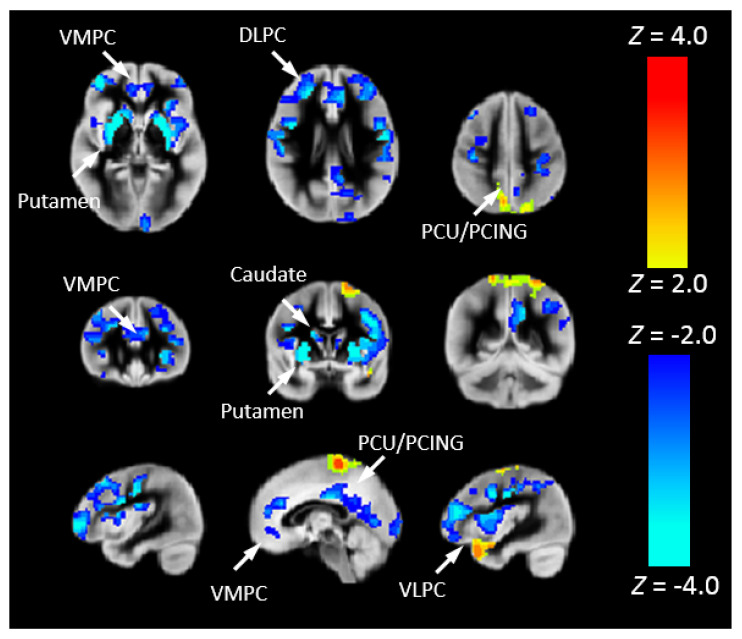
Nasal nitric oxide (nNO) mediates Pnvf in children and younger adults with CHD. Regions with a negative indirect effect (cold colors) or positive indirect effect (hot colors) with CHD status the independent variable, Pnvf the dependent variable, and nNO measurements the mediator (controlling for age and gender) are concentrated in similar regions as shown in Figure 6. All regions significant at FDR-corrected q < 0.05. Abbreviations: Ventrolateral medial prefrontal cortex (VMPC), Insula (INS), Dorsolateral prefrontal cortex (DLPC), Posterior Cingulate (PCING), Precuneus (PCU), Dorsal Anterior Cingulate Cortex (DACC), Posterior Parietal (PP).

To more closely associate Pnvf with neurobiology, we correlated Pnvf with nNO measurements in the subset of participants for which nNO measurements were available. Positive correlations of Pnvf with nNO measurements were found (Figure 6) in frontal DMN and CEN regions, with particularly strong correlations in subcortical regions (putamen). Next, we investigated whether reduced Pnvf in CHD patients is mediated by nNO. We also found a negative indirect effect (Figure 7) in these regions.

### 3.5. Demographics

Pnvf was strongly negatively correlated with participant age throughout the entire gray matter (Supplementary Material). This result is to be expected if Pnvf is related to CVR which is known to decrease throughout the lifespan. Significantly higher Pnvf was found in girls and women compared to boys and men in widespread cortical and subcortical regions not restricted to DMN/SN/CEN (Supplementary Material).

### 3.6. Test–Retest Reliability

For almost all (99.8%) voxels in the brain, R was >0.7, indicating acceptable test–retest reliability throughout the gray matter, and the R averaged over all voxels was 0.89 ± 0.041, indicating very good test–retest reliability overall (Supplementary Material).

## 4. Discussion

Nvf has been shown to play an important role in cognition, normal neural development, normal aging, and in various neuropathologies. Therefore, we hypothesized that Pnvf may not only underlie adverse neurocognitive outcomes in pediatric and young adult CHD patients but would also demonstrate associations with a proxy of nitric oxide bioavailability.

### 4.1. Proxy for Nvf

We have proposed a non-invasive MRI proxy for nvf. A cerebral biomarker, to be of optimal utility for a wide variety of research and clinical populations, should, in theory, demonstrate the following properties: (1) it must be noninvasively measurable; (2) it must not necessitate performance of a cognitive task (i.e., it can be used during the resting-state); (3) it must provide spatial coverage over the entire cortex and subcortical regions; and (4) it must not necessitate an excessively long measurement time. Our proposed metric of Pnvf provides all these properties. In fact, if an ASL acquisition and a resting-state BOLD series are already being acquired as part of the clinical or research protocol, Pnvf can be computed without any additional scan time whatsoever. Recently, another type of modeling related to combining ASL and BOLD imaging data has been recently described, leveraging other types of BOLD properties (amplitude of low frequency fluctuations) and demonstrates similar gender and neurocognitive predictions similar to our study [45].

A few previous studies have computed the ratio of CBF/FCS (the inverse reciprocal of our proposed metric) on a regionally specific basis and found it to be altered in neuropsychiatric disorders such as schizophrenia [46] and metabolic disorders such as Wilson’s disease [47] as well as decreased with healthy aging [48]. These results further support that this metric is reflective of Pnvf, which underlies cognitive activity, as higher Pnvf reflects a brain architecture which handles the task of meeting resting-state metabolic and connectivity demand more efficiently. Here we use the alternative metric obtained by computing the negative of the ratio of FCS to CBF. This choice is due to noise considerations: noise in a ratio is much more impacted by the relative error in the denominator compared to the relative error in the numerator, and the relative standard deviation of FCS is much larger than the relative standard deviation of CBF in gray matter. Since both numerator and denominator are positive definite, our biomarker is a suitable alternative for investigating between-group differences or correlations with covariates of interest with improved sensitivity.

As evidence of from our theoretical modeling, we show excellent agreement with a previously published method for estimation of the P_ET_CO2 time course from the resting-state BOLD data [33]. Our estimator was not designed to be optimal, but to rigorously test the null hypothesis of no agreement between the estimators; nevertheless, it performed at least as well as the resting-state estimator. Further research will use direct estimation of P_ET_CO2 together with resting-state BOLD acquisitions using fast techniques such as simultaneous multi-slice (SMS) echo-planar imaging [49] which can dramatically shorten the TR, and further investigate how our Pnvf metric is related to P_ET_CO2-related BOLD signal changes.

Our results also show excellent test–retest reliability, indicating that robust results may be obtained with only one BOLD resting-state acquisition. Although practical considerations (especially the likelihood of some patient motion) may dictate the need for two acquisitions to ensure sufficient data retention after motion censoring, our results indicate that reliable results may be obtained even if as much as two-thirds of the data from two runs must be discarded due to participant motion.

### 4.2. Regionally Specific Reduced Pnvf in Patients with CHD

Post-operative survival rates for CHD patients have improved dramatically in recent decades with the advancement of surgical procedures [50,51,52]. However, it has long been known that a subset of patients with CHD even without frank injury are at risk for developing cognitive problems, psychosocial dysfunction, and psychiatric disorders [7,8,9,10,11] over time. Recently, in addition, CHD adults have shown increased risk for early dementia [53]. However, the precise physiological etiology underlying these risks remains unclear. Our previous work showed that white matter structural network topology (as measured via diffusion tensor imaging) mediated poorer cognitive outcome in adolescents with transposition of the great arteries [54]. Our results of decreased Pnvf in CHD patients in DMN, SN, and CEN regions suggest that nvf is also an important factor in outcome, as CHD patients with high nvf will likely have more resiliency in response to the neurological stress related not only to CHD but also to normal stresses encountered from the environment, while CHD patients with low nvf maybe disproportionately affected. At the other end of the lifespan, our results are consistent with greater dementia risk in older CHD adults [53]. Even though our sample consisted of CHD patients who were within the normal range of neurocognitive performance on average, they performed more poorly than our control subjects. We hypothesize further reduced Pnvf in neurocognitively impaired CHD patients. The etiology of the reduced nvf is a subject for future research. It is possible that impaired substrate delivery in utero related to white matter abnormalities and brain dysmaturation [55] also affects the developing vasculature and may as well have an impact on synaptogenesis. On the other hand, various genetic etiologies have also been proposed for CHD [56,57,58], which may affect not only the developing vasculature and functional connectivity but also their functioning in older children and adults. For instance, an eNOS variant has been found more prevalent in individuals with CHD [59]; this variant would reflect in reduced Pnvf via reduced NO bioavailability.

### 4.3. Pnvf Is Associated with Neurocognitive Outcome

Pnvf also displays robust positive correlations with neurocognitive outcomes, as assessed via the NIH Toolbox Cognitive Battery, in DMN, SN, and CEN regions as well as the hippocampus. Recent studies also suggest a relationship between neurovascular integrity (as measured by CVR) and neurocognitive performance in otherwise healthy individuals across the lifespan. Memory and attention performance has been shown to be associated with regional CVR in neurocognitively healthy older adults [60]. In otherwise healthy children and adolescents with hypertension, a recent study [61] demonstrated a correlation of executive function with CVR. In general, hypertension is negatively correlated with cognitive performance in children and adolescents [62], and impaired CVR is a likely mechanism underlying hypertension [63,64,65,66,67], again suggesting a strong connection between the vasculature and optimal cognitive function.

Intriguingly, the associations found in our study population are mainly driven by the Crystallized Cognition composite as opposed to the Fluid Cognition composite. Crystallized intelligence (Gc) is experience-dependent knowledge usually related to school activities, while fluid intelligence (Gf) is the spontaneous, novel reasoning ability related to things such as pattern recognition and problem solving [68]. We therefore hypothesize that cerebrovascular reserve as estimated by Pnvf may be related to cognitive reserve (CR), a protective factor preventing or reducing cognitive impairment in response to a given brain insult. CR is a widely used concept in elderly populations involving studies of normal aging [69,70] as well as neuropathologies such as Alzheimer’s disease [69,71] and other dementias [72], and has also been used in younger adults and children with traumatic brain injury [73,74,75,76]. CR is known to correlate with lifestyle and cognitive factors, such as level of education, type of occupation, vocabulary, and frequency of reading, which are frequently referred to as “cognitive enrichment” or “intellectual enrichment” factors. Cognitive enrichment has been shown to correlate with Gc but not Gf in adolescent [77] and in elderly populations [78]. Thus, metrics which are very highly associated with Gc [such as scores from Oral-Reading Recognition Test reading (NIH Toolbox), Peabody Picture Vocabulary Test, WAIS Verbal IQ or Vocabulary subtest] are very commonly used as metrics for CR [79,80,81], in combination with educational, occupational, and social measures.

This hypothesis leads to the intriguing possibility that nvf may be modifiable in CHD patients, thus significantly reducing the risk of adverse neurocognitive outcome via appropriate behavioral interventions and preventive strategies. A similar strategy (e.g., modifying CR via cognitive enrichment) has been previously successfully utilized in Alzheimer’s and dementia patients [70,82]. This possibility is supported by recent research into the neurobiological underpinnings of Gc. A genome-wide association study found that genetic-related underlying factors such as quantity and morphology of neurons and synapses are mainly responsible for Gf whereas genetic-related underlying synaptic fatigue and long-term depression mainly underlie Gc [83], consistent with long-term neuroplasticity being more influential for Gc (as it is experience-dependent) as compared to Gf. Short-term plasticity, such as produced by synaptic fatigue, is also relevant as it has been shown to be important in expanding the range of long-term plasticity [84]. The link between nvf and Gc may be partially explained by astrocytes, which regulate vascular tone in the resting-state [85]; additionally, astrocytes connect with synapses and influence synaptic plasticity (hence the concept of the “tripartite synapse” [86]). One of the mechanisms involved is astrocytic mediation of heterosynaptic LTD [87]; astrocytes have likewise been shown to mediate short-term synaptic fatigue [88]. The communication between neurons and astrocytes is bi-directional [86,89,90,91] with dynamic changes in astrocytes due to synaptic activity, supporting the possibility that nvf may be experience-dependent and modifiable through astrocytic mediation. Astrocytes may also be directly related to neurological outcome in CHD, as astrocyte dysfunction may underlie various neurological diseases throughout the lifespan [92,93]. Interestingly, NO is also produced via eNOS in astrocytes [94], corresponding to the genetic variant prevalent in CHD patients cited above [59].

### 4.4. Reduced Pnvf Mediates Adverse Neurocognitive Outcome in CHD Patients

Our mediation results provide further support for our hypothesized framework of reduced nvf in CHD patients partially underlying the risk of adverse neurocognitive outcome. Mediation analyses provide more powerful and informative tests for causality compared to simple correlations when direct manipulation of independent variables and mediators is not feasible [35], although only such manipulation can provide proof in the strict sense, such as findings obtained from a randomized clinical trial. Here, we found a negative indirect effect in DMN, SN, and CEN regions of Pnvf on the relation between CHD status and neurocognitive outcome (Total Cognition composite); and since the CHD subjects had lower Total Cognition scores compared to the normal referents, we conclude that reduced Pnvf mediates adverse neurocognitive outcome in this population.

### 4.5. Demographic Considerations

Through the developmental period up until the onset of aging, girls and women are in general more resilient to neurologic insult, with lower incidence of neuropathologies including autism spectrum disorder [39], ADHD [40,41], schizophrenia [42], intellectual disability [43], and dyslexia [44]. Our results here suggest that increased nvf in girls and women may play a significant role in this phenomenon, although further research will be necessary to investigate this hypothesis in more detail. The difference is particularly pronounced in CEN regions, which may relate to boys’ and men’s greater vulnerability to executive function disorders such as ADHD. Since CBF also decreases with age and is higher in girls and women throughout the lifespan [95], especially after puberty and in orbitofrontal and temporal regions, future research will be needed to determine whether Pnvf or CBF alone is a better indicator of physiological age-related changes and gender differences related to differential risk for various neuropathologies.

### 4.6. Relations with nNO Levels

In a subset of these participants, nNO measurements were obtained. Low nNO is a biomarker for poor vascular endothelial health and impaired cardiac function in pediatric CHD patients [7]. Cerebral NO is a neurotransmitter [96,97,98] generated in synapses and blood vessels [96] subsequent to neuronal activity and is also important for autoregulation [8,9,10]. Our ancillary analysis found widespread positive correlations between Pnvf and nNO. Thus, we suggest a biochemical basis of Pnvf due to NO bioavailability. This is further supported by our mediation results, as we also saw a negative indirect effect for nNO mediating the relation between CHD and Pnvf in DMN and CEN regions.

NO in the nasal cavity is primarily produced via inducible NO synthase (iNOS) [99] present in the upper airway and paranasal sinuses, while cerebral NO is primarily produced via neuronal NO synthase (nNOS) and endothelial NO synthase (eNOS) present in the cerebral vasculature [96]. However, several plausible biological mechanisms [100] could account for decreased cerebral NO bioavailability together with decreased nNO in CHD patients. NO is produced via the conversion of L-arginine to L-citrulline. Thus, one possible explanation is decreased overall availability of L-arginine, possibly due to higher levels of arginase (which also has L-arginine as a substrate); decreased L-arginine bioavailability has been shown in cystic fibrosis (CF) patients [101], who also display lower levels of nNO. In fact, pilot studies have shown increased nNO levels in primary ciliary dyskinesia (PCD) and CF patients following L-arginine administration [102], raising the possibility that L-arginine administration may also be of clinical benefit in the CHD population. Alternatively, tetrahydrobiopterin (BH4) bioavailability [103] may underlie our results, as BH4 is a cofactor for all isoforms of NOS. Not only will reduced BH4 bioavailability reflect in lower overall rates of synthesis of NO, but the reaction itself may become “uncoupled” from L-arginine oxidation, resulting in the production of O2^−^ instead of NO [104], a reaction which is implicated in a variety of vascular disease states [105,106]. In fact, some preliminary results suggest BH4 administration may improve vascular function in hypertension, atherosclerosis, and other vascular disorders [103]. Finally, a genetic etiology cannot be ruled out; while different genes are involved in iNOS expression and eNOS expression, there is the possibility of linkage disequilibrium between those genes.

### 4.7. Limitations

While our Pnvf metric is not intended to be a substitute for CVR, it will be of interest to investigate the relation between Pnvf and CVR estimated via other methods such as a hypercapnic challenge. Furthermore, it should be noted our metric is not specific to neuronal metabolic demand; there is significant oxidative metabolism in glial cells, in particular astrocytes. Additionally, we did not adjust for hematocrit, and further research will investigate whether appropriate adjustments for hematocrit will result in an even better proxy for nvf.

An important limitation of our study is that we did not obtain concurrent measurements of oxygen saturation and hematocrit/hemoglobin. As such the impact of oxygen saturation and polycythemia on our Pnvf metric is unknown and could not be controlled for in this study. Additionally, recruitment of CHD patients with heterogenous heart lesions may compound this limitation, even though we did note that single ventricle cardiac patients in our sample did demonstrate more profound deficits in neurovascular function compared to bi-ventricular carsiac lesion and controls. We also note that our cardiac sample did not include transposition cardiac lesion cases. In addition, because our patients were recruited cross-sectionally, important longitudinal clinical data including number of surgeries and technical details of the surgeries were not available for all CHD patients enrolled in our study.

We also acknowledge that CBF (of our FCS/CBF measurement) is multifactorial, especially with regard to key variables that may be different within the CHD population. In particular, vascular resistance, cardiac output, SpO_2_, and hemoglobin exist in a flux necessary to provide adequate cerebral oxygenation. Given oxygen delivery is the product of cardiac output and arterial oxygen content, cyanotic CHD patients will require either a higher cardiac out (i.e., flow) or higher hemoglobin (to increase content). These patients will not overcorrect, but as they become more desaturated and/or develop worsening cardiac output, they develop polycythemia to compensate. While this compensation maintains adequate oxyhemoglobin concentrations, it does result in increased deoxyhemoglobin. Excess NO becomes oxidized to nitrite and nitrate, which drastically increases its circulating half-life and any excess is removed in the urine. However, given desaturated CHD patients have more deoxyhemoglobin as a result of their compensatory polycythemia, they are more readily reducing nitrate/nitrite so they have less circulating storage forms of NO in order to maintain an NO balance. Thus, since NO can merge between three different forms based on need, we believe that what is measured from the nares is reflective of systemic NO availability, given those who require increased reduction of nitrate/nitrite to molecular NO still maintain NO levels but do not have luxury NO to spare.

Our single-slice 2-D EPI acquisitions for ASL and BOLD, while having the advantage of being straightforward to implement on most scanner platforms, are not fully optimal. The BOLD acquisition would benefit from a simultaneous multi-slice (SMS) EPI readout [49], which would greatly speed up the TR (by a factor of 4×−6× or more depending on coil geometry and SMS factor), enabling acquisition of many more frames for the same total acquisition time and hence significantly improved CNR. The ASL acquisition would have benefitted from background suppression pulses [107,108], which can improve CNR by suppressing background signal from the parenchyma, and can be implemented using a 3D acquisition such as GRASE [107,109] or stack of spirals [110,111], although improving the ASL acquisition will not have as much impact in overall CNR compared to FCS as the noise in our Pnvf metric is predominantly driven by noise in the FCS estimator. Nevertheless, our non-optimal sequences provide a “worst-case” or “typical-case” scenario for test–retest reliability, which is very good even with the sequences used in this study, and indicates Pnvf can be reliably estimated even with typically available pulse sequences.

Our 2D GE-EPI ASL readout is also limited in that a tradeoff is necessary in selecting the initial (e.g., for the first acquired slice) pCASL post-labeling delay. A shorter delay results in higher ASL signal contrast between control and labeled acquisitions and hence greater SNR; however, the two-compartment model is more sensitive to misestimations of tissue transit time for absolute CBF estimation [26]. Thus, our initial post-labeling delay is somewhat shorter than that recommended by the recent pCASL white paper [108], although its suggested value was for adult subjects and not for older children, who have slightly shorter transit times. However, the actual post-labeling delay increases as a function of slice position (ascending through the brain), so that by the time the level of the basal ganglia (approximately the 10th slice) is reached, the true post-inversion delay is around 1600 ms and close to that recommended by the white paper, as given the time necessary for fat saturation, excitation, etc., the total acquisition time for each slice is around 40 ms. Thus, it is possible that, for regions in the lower portion of the brain, our results may have been somewhat affected by transit time effects. However, most of our significant results were at the level of the basal ganglia or higher. In future studies, if a 3D acquisition is not employed, a 2D SMS readout [112,113] may be used to greatly shorten the total acquisition time and optimize the post-inversion delay for all slices without significant SNR loss.

Finally, we used a similar method of computing FCS maps used here as in previous work [21] which involves setting all negative correlation coefficients to zero prior to taking the average. However, other studies [46,47] have used different thresholds (0.2 or 0.25), setting all subthreshold correlation coefficients to zero. The precise effect of the specific threshold used is at present unknown and remains a subject for future research. Additionally, the functional connectivity was restricted to positive correlation coefficients, whereas it is known that strong and relevant anti-correlations exist, for instance between the DMN and the CEN in adults and adolescents [114,115]. An alternative version of computing FCS, to be investigated in the future, could use the absolute values instead of the signed values of the correlation coefficients, with or without a threshold.

## 5. Conclusions

An MRI proxy for neurovascular function (Pnvf) is proposed which may be obtained noninvasively in the resting-state as the negative of the ratio of FCS to CBF. Pnvf is shown to be reduced in CHD subjects, especially in the DMN, CEN, and SN. Additionally, Pnvf positively correlates with neurocognitive outcome and reduced Pnvf mediates worse neurocognitive outcomes and is associated with nitric oxide bioavailability in pediatric and adult CHD patients. Our findings suggest that neurovascular function combined with nitric oxide availability measures have the potential to identify CHD patients who are at the highest risk of neurocognitive deficits. These results provide evidence that impaired nitric oxide dependent nvf may partly underlie adverse neurocognitive outcomes in CHD. These findings also support the concept that interventions geared toward improving nitric oxide bioavailability, have the potential to improve neurocognitive outcomes in patients with CHD.

## Figures and Tables

**Table 1:** Demographic and cognitive characteristics (NIH Toolbox scores) for the study population, consisting of subjects with congenital heart disease (CHD) and normal referents, as well as nasal nitric oxide measurements and maternal education levels for the subset of participants where those data were available (44 for nasal nitric oxide, 71 for maternal education). Maternal education was coded according to the following rubric: Doctoral/Professional degree = 7, Master’s degree = 6, Bachelor’s degree = 5, Associate’s degree = 4, Trade/technical/vocational training = 3, Some college = 2, HS graduate = 1, No HS diploma = 0.

	CHD	Controls	*p*
N	24	64	
Age (years)	15.8 +/− 3.64	14.0 +/− 3.61	0.036
Gender	10 F, 14 M	32 F, 32 M	0.486
Maternal Education	4.06 +/− 1.73	4.73 +/− 1.92	0.198
Nasal Nitric Oxide (nL/min)	240.5 +/− 105.72	283.7 +/− 83.61	0.138
NIH TOOLBOX			
Total Cognition Composite	107.1 +/− 16.21	116.6 +/− 22.02	0.062
Crystallized Cognition Composite	109.3 +/− 11.67	115.6 +/− 16.25	0.091
Picture Vocabulary	103.8 +/− 11.28	108.2 +/− 14.33	0.194
Oral Reading Recognition	111.2 +/− 15.13	117.8 +/− 17.09	0.107
Fluid Cognition Composite	103.0 +/− 17.44	109.5 +/− 20.78	0.186
Dimensional Change Card Sort	100.5 +/− 16.97	104.2 +/− 16.17	0.361
Pattern Comparison	104.0 +/− 23.24	107.5 +/− 23.35	0.537
Picture Sequence Memory	101.7 +/− 18.18	108.1 +/− 17.97	0.148
List Sorting	104.9 +/− 15.00	107.0 +/− 14.50	0.564
Flanker	98.9 +/− 11.81	100.8 +/− 15.02	0.573

**Table 2:** Types of congenital heart defects in the study population (*N* = 24).

Hypoplastic left heart syndrome	5
Other single ventricle defects	5
Biventricular left heart obstructive lesions (Aortic stenosis and/or coarctation)	7
Tetralogy of Fallot variants	4
Septal defects (Ventricular and atrioventricular)	3

## Data Availability

Data is available upon written request to corresponding author.

## Supplementary Materials

The following supporting information can be downloaded at: https://www.mdpi.com/article/10.3390/metabo12090882/s1, Supplemental Figure S1. Comparison of Pnvf as a function of age between CHD (red) and controls (blue) in specific regions shown in Figure 3: (A) dorsolateral prefrontal region; (B) insular cortex; (C) anterior cingulate; and (D) posterior cingulate/precuneus; indicating that the Pnvf differences are more prevalent at older ages. Supplemental Figure S2. Pnvf positively correlates with neurocognitive outcomes in children and younger adults with CHD and normal referents. Regions with a positive correlation of Pnvf with the NIH Toolbox Crystallized Cognition composite score (controlling for age, gender, and CHD status) are mainly concentrated in DMN, SN, and CEN regions as well as hippocampus and putamen. All regions significant at FDR-corrected q < 0.05. Supplemental Figure S3. Reduced Pnvf mediates adverse neurocognitive outcome in children and younger adults with CHD. Regions with a negative indirect effect with CHD status the independent variable, NIH Toolbox Crystallized Cognition Composite score the dependent variable, and Pnvf the mediator (controlling for age and gender) are mainly concentrated in DMN, SN, and CEN regions. All regions significant at FDR-corrected q < 0.05. Supplemental Figure S4. Pnvf decreases with age. Pnvf negatively correlates with age (left; controlling for gender and CHD status) throughout the cerebrum. All regions significant at FDR-corrected q < 0.05. Supplemental Figure S5. Pnvf is greater in girls and women. Pnvf is greater in girls and women compared to boys and men (right; controlling for age and CHD status) throughout the cerebrum. All regions significant at FDR-corrected q < 0.05. Supplemental Figure S6. Pnvf provides excellent test–retest repeatability. Histogram of test-retest repeatability for all voxels in the global gray matter mask. Average R = 0.89 ± 0.041, demonstrating excellent repeatability throughout the gray matter.
