# Supplementary figures and images for: Impaired Neurovascular Function Underlies Poor Neurocognitive Outcomes and Is Associated with Nitric Oxide Bioavailability in Congenital Heart Disease
Source: Metabolites. 2022 Sep 19;12(9):882. doi: 10.3390/metabo12090882 (PMC9504090; doi:10.3390/metabo12090882)

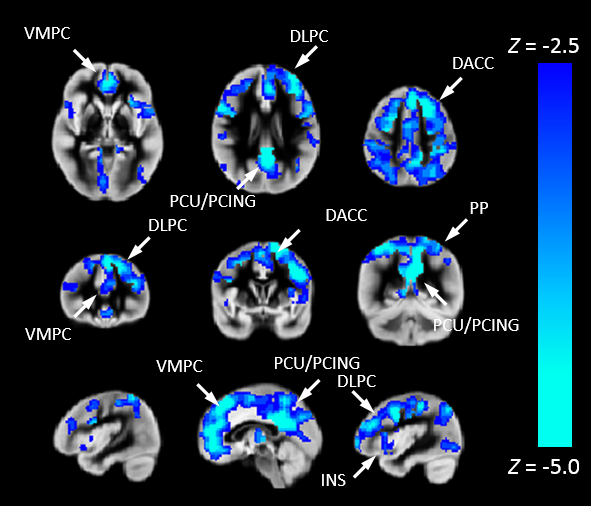

Supplement: Supplementary file 1 [file metabolites-12-00882-s001.zip › supplementary figures/Supplementary Figure S1.png]

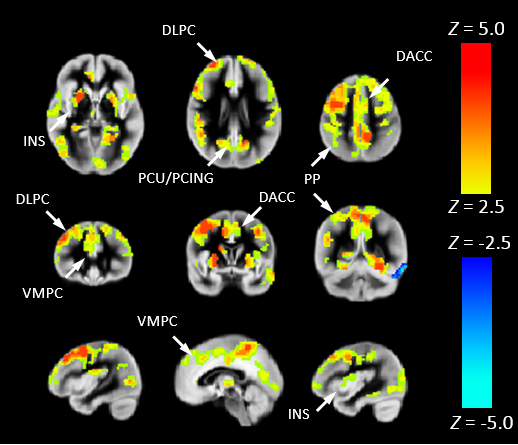

Supplement: Supplementary file 1 [file metabolites-12-00882-s001.zip › supplementary figures/Supplementary Figure S2.png]

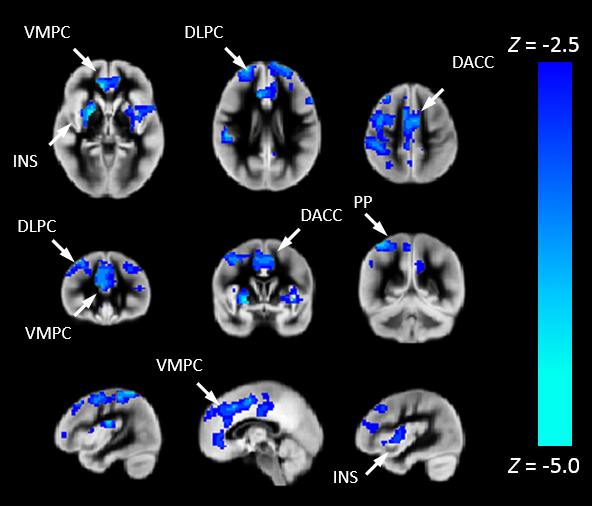

Supplement: Supplementary file 1 [file metabolites-12-00882-s001.zip › supplementary figures/Supplementary Figure S3.png]

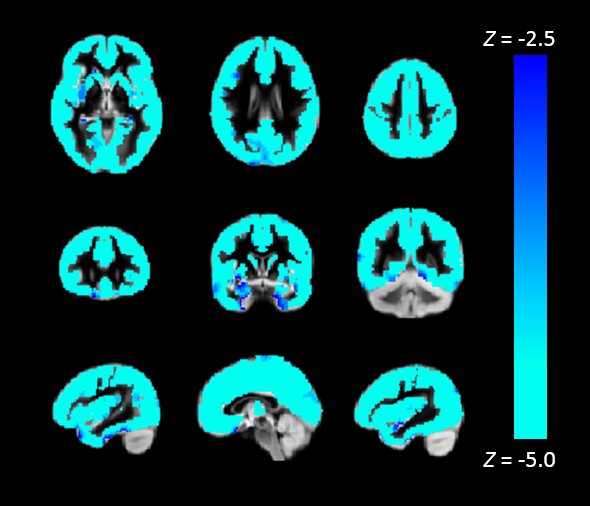

Supplement: Supplementary file 1 [file metabolites-12-00882-s001.zip › supplementary figures/Supplementary Figure S4.png]

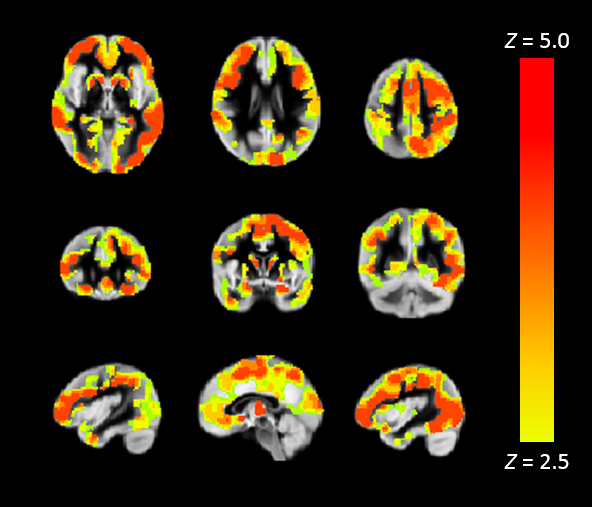

Supplement: Supplementary file 1 [file metabolites-12-00882-s001.zip › supplementary figures/Supplementary Figure S5.png]

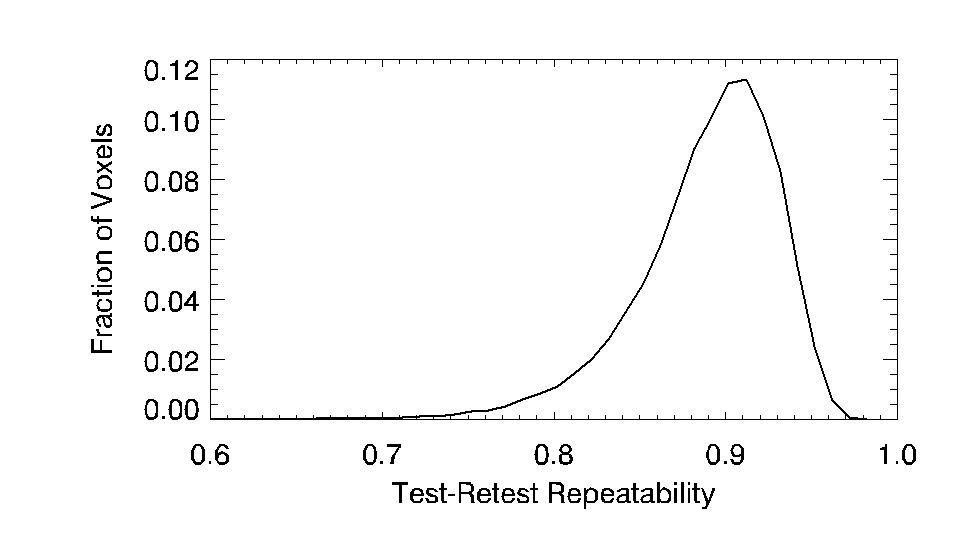

Supplement: Supplementary file 1 [file metabolites-12-00882-s001.zip › supplementary figures/Supplementary Figure S6.png]
